# Seasonal Infective Dynamics and Risk Factors Associated with Prevalence of Zoonotic Gastrointestinal Parasites from Meat Goats in Southern Thailand

**DOI:** 10.3390/ani15142040

**Published:** 2025-07-11

**Authors:** Narin Sontigun, Chalutwan Sansamur, Tunwadee Klong-Klaew, Morakot Kaewthamasorn, Punpichaya Fungwithaya, Raktham Mektrirat

**Affiliations:** 1 Office of Administrative Interdisciplinary Program on Agricultural Technology, School of Agricultural Technology, King Mongkut’s Institute of Technology Ladkrabang, Bangkok 10520, Thailand; narin.so@kmitl.ac.th (N.S.); punpichaya.pu@kmitl.ac.th (P.F.); 2 Akkhraratchakumari Veterinary College, Walailak University, Nakhon Si Thammarat 80160, Thailand; chalutwan.sa@wu.ac.th; 3 Department of Parasitology and Entomology, Faculty of Public Health, Mahidol University, Bangkok 10400, Thailand; tunwadee.klo@mahidol.edu; 4 Center of Excellence in Veterinary Parasitology, Department of Pathology, Faculty of Veterinary Science, Chulalongkorn University, Bangkok 10330, Thailand; morakot.k@chula.ac.th; 5 Veterinary Academic Office, Faculty of Veterinary Medicine, Chiang Mai University, Chiang Mai 50100, Thailand; 6 Veterinary Research Center for Veterinary Biosciences and Veterinary Public Health, Faculty of Veterinary Medicine, Chiang Mai University, Chiang Mai 50100, Thailand; 7 Center of Excellence in Pharmaceutical Nanotechnology, Faculty of Pharmacy, Chiang Mai University, Chiang Mai 50200, Thailand

**Keywords:** biodiversity, caprine, coccidia, helminths, internal parasites, tropical climate, strongyles, surveillance, zoonotic diseases

## Abstract

Gastrointestinal (GI) parasites not only significantly impact goat health and productivity but can also affect human health due to the zoonotic potential of some species. This study investigates the prevalence of internal parasites within the tropical monsoon ecosystem of southern Thailand, focusing on both phenotypic and molecular characteristics of the parasites and identifying associated risk factors in caprine farming systems. A total of 276 meat goats from Nakhon Si Thammarat province were examined, indicating an overall GI parasite prevalence of 88.8% (245/276), with strongyles and *Eimeria* spp. identified as the dominant parasites. In addition, mixed parasitic infections were observed in 72.2% of cases, whereas single infections comprised 27.8%. Strongyle-positive fecal samples were cultured and genetically sequenced, revealing the presence of *Haemonchus contortus*, *Trichostrongylus colubriformis*, and *Oesophagostomum asperum*. For associated risk factors, gender and grazing with other herds significantly impacted overall GI parasitic infections, while the gender, breed, and packed cell volume (PCV) affected the strongyle infection. A correlation analysis revealed a substantial relationship between strongyle egg per gram (EPG) counts and clinical parameters, indicating that monitoring animals with low body condition scores (BCS) and high Faffa Malan Chart (FAMACHA) scores could be an effective strategy for controlling strongyle infections. These findings highlight the importance of continued research and effective farm management practices to address strongyle infections in meat goats, improving their health and agricultural productivity in tropical regions. Moreover, the detection of four zoonotic parasites (*Giardia* spp., *H. contortus*, *T. colubriformis*, and *Fasciola* spp.) indicates the necessity for the routine surveillance and monitoring of zoonotic parasites in goats to mitigate potential human health risks.

## Simple Summary

This study explores the presence and risk factors of harmful parasites in goat farming within a tropical monsoon climate in southern Thailand. An examination of 276 goats revealed that 88.8% were infected with various parasites. The most common types were strongyles and coccidia, with many goats having multiple infections at once. Fecal cultures of strongyle-positive samples, and DNA sequencing, confirmed the presence of three parasite species: *Haemonchus contortus*, *Trichostrongylus colubriformis*, and *Oesophagostomum asperum*. Factors such as the goats’ gender and whether they graze with other herds affect the likelihood of these infections. Additionally, monitoring goats that appear thin and have high anemic scores can help manage and reduce these infections. This research is crucial for improving the health and productivity of meat goats, ultimately benefiting farmers and the agricultural community.

## 1. Introduction

Goats are raised globally to fulfill various purposes, including nutritional, economic, and socio-cultural needs, mainly in Africa and Asia [1]. In the tropical monsoon climate of Thailand, the southern region accounted for 29.5% of the national goat population in 2022, making it the second-largest area for goat raising. This follows the central region, which held 34.9% of the total goat population [2]. Most goats in the southern regions are meat goats, typically raised using five systems: tethering (tethering goats on public and arable lands), extensive (allowing goats to graze freely on public land), semi-intensive (allowing goats to graze in pastures during the day and housing them indoors at night), intensive (keeping goats in pens full-time and feeding them roughage, such as grasses and oil palm fronds, along with concentrate feeds), and livestock–tree crop integration (rearing goats under tree crops in areas used for other ruminant production) [3,4]. Goats that graze on pastures or are subjected to inadequate farm management practices are more likely to acquire gastrointestinal (GI) parasitic infections. These infections can cause stunted growth, reduced production, and, in severe cases, death, resulting in substantial economic losses for goat farmers. GI parasitic infections in goat herds can cause mortality rates of over 40% and annual weight losses ranging from 6 to 12 kg per animal [5]. A high prevalence of GI parasitic infections in goats has been reported in several regions of Thailand, including the northern (87.2%), the central (68.65–96.37%), the northeast (100%), and the southern (75.2–97.33%) regions [6,7,8,9,10,11,12,13,14,15,16], with the majority of goats infected with multiple parasite species. Furthermore, goats act as reservoir hosts for various zoonotic protozoans (e.g., *Cryptosporidium* spp., *Giardia* spp., and *Toxoplasma gondii*) and zoonotic helminths (e.g., *Haemonchus contortus*, *Trichostrongylus* spp., *Echinococcus* spp., *Fasciola* spp., *Eurytrema pancreaticum*, and *Dicrocoelium dendriticum*) that can be transmitted to humans [17,18,19,20]. Given the public health significance of GI parasites in goats, personal hygiene promotion and effective parasite control in goats are critical to reducing environmental contamination. The routine surveillance and monitoring of GI parasites should be implemented, and infected animals should be treated immediately to minimize the parasitic burden and transmission.

In Nakhon Si Thammarat province, research on GI parasites in caprine farming has been limited, with only one study completed in the past decade by Worasing et al. [7], resulting in insufficient and outdated data on GI parasites in meat goats. Therefore, this study aims to investigate the prevalence and risk factors linked to GI parasites in meat goats in Nakhon Si Thammarat province, southern Thailand. This study not only provides essential data for establishing effective parasite prevention and control strategies in goats but also offers a basis for the assessment of zoonotic risks to human health.

## 2. Materials and Methods

### 2.1. Animals and Ethical Statement

Based on the Epi Info^TM^ software version 7.2.5.0 (Centers for Disease Control and Prevention, Atlanta, GA, USA), using a goat population of 48,181 in Nakhon Si Thammarat with an expected frequency of 90%, a precise error of 5%, clusters of 12, and a confidence level of 95%, the estimated sample size was 144 goats. The research procedures detailed in this study were approved by the Institutional Animal Care and Use Committee of Walailak University (Approval number: WU-ACUC-66033).

### 2.2. Study Areas and Sampling

**Figure 1:**
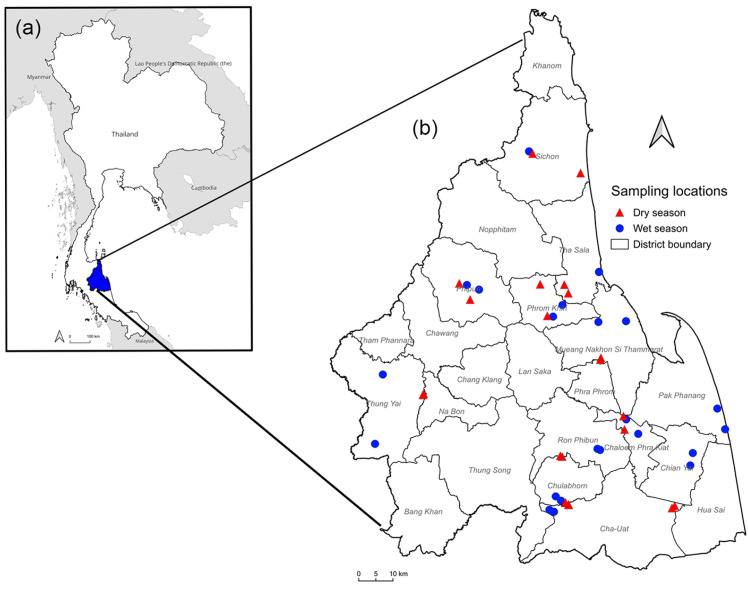
A map showing the sample collection sites in Nakhon Si Thammarat, southern Thailand. (**a**) A map of Thailand with Nakhon Si Thammarat province highlighted in blue. (**b**) Surveyed locations of 46 farms across 12 districts within Nakhon Si Thammarat.

According to the Thai Meteorological Department, the climate in southern Thailand is characterized by two distinct seasons: the wet season, occurring from June to January, and the dry season, extending from February to May. The present study was conducted from June 2023 to April 2024. Additionally, two meat goat farms in 12 districts were randomly selected (Figure 1) to be study sites (8°26′6.59″ N 99°57′28.19″ E). In each season, six goats were randomly selected from each of the 24 farms using simple random sampling, yielding a total of 144 goats per season. Therefore, 288 fecal and blood samples from meat goats in Nakhon Si Thammarat province were used in this study. Due to the low market price of goats during the dry season, many farmers sold their livestock owing to an inability to afford production costs, leading to many leaving the goat farm industry. The QGIS version 3.40.3 software (https://qgis.org/; accessed on 10 February 2025) was utilized to map the sampling locations. Boundary data for Thailand, its neighboring countries, and Nakhon Si Thammarat province, obtained from the Humanitarian Data Exchange (HDX) (https://data.humdata.org; accessed on 10 February 2025), was incorporated to generate the map.

### 2.3. Farm Data and Health Assessment

The breed, gender, and age were recorded from various sources. Additionally, data on farm management (intensive or semi-intensive), water source nearby (yes or no), grazing with other herds (yes or no), grazing rotation (yes or no), deworming interval (≤6 months or >6 months), and the number of drug types used per year (one type or two types) were collected through interviews with the farmers. The body condition score (BCS) and conjunctival coloring assessed by the FAMACHA^©^ score for anemia were recorded. Breeds were classified as pure or mixed. The age of the goats was recorded and categorized into ≤2 years and >2 years. The BCS was classified into two categories: poor (BCS ≤ 2) and good (BCS > 2), according to the 1–5 scale defined by Ghosh et al. [21]. FAMACHA scores were allocated according to conjunctival coloration, following the classification established by van Wyk and Bath [22], as follows: 1 = red (non-anemic), 2 = red–pink (non-anemic), 3 = pink (mildly anemic), 4 = pink–white (anemic), and 5 = white (severely anemic).

### 2.4. Fecal and Blood Collections

A fecal sample was collected directly from the rectum of individual animals and placed in a plastic Ziplock bag. In addition, a 3 mL blood sample was taken from the jugular vein of individual animals and immediately transferred into a 3 mL vacuum blood tube containing ethylenediaminetetraacetic acid (EDTA). All samples were kept in an icebox and transported to the parasitology laboratory at Akkharatchakumari Veterinary College, Walailak University, where samples were stored at 4 °C until analysis. Furthermore, the packed cell volume (PCV) value was investigated by a microhematocrit method for anemia detection. Briefly, the EDTA blood was filled in a capillary tube, sealed with modeling clay, and centrifuged at 11,500–15,000 rpm for 5 min, and then the result was read using a hematocrit reader. Normal PCV values for goats range from 22% to 38%; animals with lower PCV values are considered anemic [23]. Two hematocrit measurements were performed on each blood sample, and the mean of these two measures was calculated.

### 2.5. Microscopic Examination of Fecal Samples

Fecal samples were analyzed using both qualitative and quantitative methods. Simple flotation and sedimentation techniques [24] were carried out to detect the presence of GI parasites, i.e., helminth eggs and protozoal cysts or oocysts in fecal samples. The presence of parasites was observed by their morphological characteristics under light microscopy (Nikon, Melville, NY, USA) using 10× and 40× objective lens. For positive samples, fecal egg counts (FEC) and oocyst counts (FOC) were examined using the modified McMaster technique with a sensitivity of 50 eggs/oocysts (EPG/OPG) of feces [25]. According to Soulsby [24], FEC values of strongyle-type eggs were classified into negative, low (<500 EPG), medium (500–1000 EPG), and high (>1000 EPG). Similarly, FOC values were classified into negative, low (<1800 OPG), medium (1800–6000 OPG), and high (>6000 OPG), according to Idris et al. [26].

### 2.6. Fecal Culture and Larval Identification

Because strongyle nematodes (*Cooperia* spp., *Haemonchus* spp., *Oesophagostomum* spp., and *Trichostrongylus* spp.) have similar egg appearances and are difficult to differentiate from each other, fecal cultures were conducted by pooling positive samples of individual farms to determine the specific nematode genera involved. Third-stage larvae (L3) were recovered from the fecal culture using the Baermann technique [25]. Subsequently, 100 randomly selected L3 from each farm were examined with a light microscope (Nikon, USA) and identified to the genus level following the morphological key of van Wyk and Mayhew [27]. When fewer than 100 L3 were isolated from a sample, the percentage of larval types was calculated based on the counted L3. The L3 were exsheathed using 0.187% sodium hypochlorite for 3–5 min [28] and washed with distilled water three times prior to use for molecular analysis.

### 2.7. Molecular Identification of Strongyle Nematode Eggs

Molecular identification of strongyle nematode eggs was performed to identify the genera. Strongyle egg-positive fecal samples from each farm were pooled, with 1 g of feces from each positive goat on each farm used. Eggs were subsequently isolated from the pooled samples using the flotation technique. The egg suspension was centrifuged at 1500× *g* for 5 min. The resultant sediment was resuspended in 10 mL of sterile water and then centrifuged at 1500× *g* for 5 min. The resuspension and centrifugation process was performed twice. After three cycles of resuspension and centrifugation, the resultant fecal pellet was resuspended in 0.5 mL of sterile water, transferred to a microcentrifuge tube, and subjected to centrifugation at 5500× *g* for 5 min. The supernatant was carefully removed by pipetting, and the residual pellet was used for DNA extraction. Genomic DNA from individual farm samples was extracted using the SimpleWay™ gDNA Prep and PCR Set I Kit (Biofact, Daejeon, Republic of Korea), according to the manufacturer’s protocol. Briefly, eggs were disrupted by adding 100–200 µL of SLB buffer, 5 µL of Proteinase K, and stainless-steel beads into each 2 mL tube, followed by processing in a TissueLyser LT apparatus (Qiagen, Hilden, Germany; Cat No./ID: 85600) at 50 Hz for 5 min. Subsequently, the tubes were placed in a heat block at 99 °C for 10 min, allowed to cool for 2 min, and centrifuged at 12,879× *g* for 1 min. The supernatant was transferred to a new tube and stored at −20 °C until further analysis. Semi-nested PCR was performed to detect the presence of strongyles in the fecal samples, utilizing primers targeting regions of strongyle nematode ribosomal DNA and ITS2, as previously described (Table 1) [13].

PCR reactions contained 6.25 μL of PCR master mix III (2×) (Biofact), 1–2 μL of DNA template, 0.2 μM of each forward (Strongyle F2) and reverse (Strongyle R3) primer (Table-1), and nuclease-free water to a final volume of 12.5 μL. The PCR cycle included an initial denaturation at 95 °C for 2 min, followed by 35 cycles of denaturation at 95 °C for 20 s, annealing at 50 °C for 30 s, extension at 72 °C for 30 s, and a final extension at 72 °C for 5 min. The amplified PCR products were separated by electrophoresis on a 1.5% agarose gel in 1 × Tris–acetate–EDTA buffer, stained with SERVA DNA Stain G (Serva, Heidelberg, Germany), and visualized under UV light using the ChemiDoc™ Imaging System (Bio-Rad, Hercules, CA, USA). After the first PCR cycle, the semi-nested PCR reaction and thermal cycling conditions were performed as described above, except the reverse primer was replaced with a genus-specific primer (Table 1), and 1 µL of the diluted F2/R3 PCR product (1:50 in nuclease-free water) was used as the template.

### 2.8. Molecular Identification of Strongyle Nematode L3

To confirm the genus of *Haemonchus*, *Oesophagostomum*, and *Trichostrongylus* identified based on morphological characteristics from each farm, three representative L3 from each genus were individually pooled. DNA was extracted using the SimpleWay™ gDNA Prep and PCR Set I Kit (Biofact, Republic of Korea) according to the manufacturer’s protocol, which was similar to the previously described method except for the use of 50 µL of SLB buffer. To identify the species of parasites, PCR-positive products obtained by Strongyle F2 and Strongyle R3 primers were purified with the E.Z.N.A.^®^ Cycle Pure Kit (Omega Bio-Tek, Norcross, GA, USA) following the manufacturer’s instructions and subsequently sent to U2Bio Sequencing Service Co., Ltd. (Bangkok, Thailand) for Sanger sequencing. The obtained sequences were manually edited and assembled into complete bidirectional consensus sequences using BioEdit software version 7.2.5 [29]. All sequencing results were compared with sequences available in the GenBank database using the Basic Local Alignment Search Tool (BLAST) version 2.16.0+ (https://blast.ncbi.nlm.nih.gov; accessed on 20 September 2024) for species identification. Furthermore, the maximum likelihood tree was reconstructed using the Tamura 3-parameter with a gamma distribution model, the best-fit nucleotide substitution model for the dataset, and was tested using 1000 bootstrap replications in MEGA X software (version 10.2.6) [30]. For nucleotide sequence accession numbers, a total of 21 strongyle sequences obtained in this study were deposited in the GenBank database under the following accession numbers: PQ358817–PQ358824 for *Haemonchus contortus*, PQ358825–PQ358834 for *Trichostrongylus colubriformis*, and PQ358835–PQ358837 for *Oesophagostomum asperum*.

### 2.9. Statistical Analysis

To assess the associations between the positivity of GI and strongyle parasites in meat goat farms and potential risk factors (season, breed, gender, age, BCS, PCV, FAMACHA score, farm management, water source nearby, grazing with other herds, grazing rotation, deworming interval, and the number of drug types used per year), both univariable and multivariable logistic regression models were assessed using R statistical software (version 4.1.3) [31], employing the ‘stats’ [31] and ‘rms’ packages [32]. Univariable logistic regression analysis was conducted for each variable, determining odds ratios and 95% confidence intervals. Variables with a *p*-value < 0.2 were included in the multivariable logistic regression model. The ‘gmodels’ package [33] was used to evaluate categorical risk factors to prevent multicollinearity. In cases of observed multicollinearity (*p* < 0.05), the variable with greater biological significance was retained. Model selection was informed by the Akaike Information Criterion (AIC), establishing an optimal equilibrium between model complexity and explanatory power. The resultant model’s goodness-of-fit was assessed using the Hosmer–Lemeshow test [34] to determine its adequacy in representing the observed data. The multivariable logistic regression model was specified using the following equation:

$$In\;\left(\frac{P_{i}}{1-P_{i}}\right)=\beta_{0}+\beta_{1}X_{1}+\ldots +\beta_{k}X_{k}$$

where *P_i_* represents the positivity of GI or strongyle parasites in meat goat farms on animal *i* (*i* = 1, …, 276), *X_k_* denotes a set of risk factors (*X_k_* = 1, …, *k*), and *β_k_* corresponds to the estimated coefficient for each respective risk factor (*β_k_* = 1, …, *k*).

Pearson’s Chi-square test was conducted to assess the association between the infection intensity of strongyle EPG, *Eimeria* OPG counts, and season. Spearman’s rank correlation coefficient (*r_s_*) was used to evaluate the relationships between BCS, FAMACHA score, and PCV with strongyle egg counts and the relationship between FAMACHA score and PCV. Statistical analyses, including Pearson’s Chi-square test and Spearman’s rank correlation, were performed using IBM SPSS statistics Software version 29.0 (IBM, Armonk, NY, USA), with statistical significance set at *p* < 0.05.

## 3. Results

### 3.1. Prevalence of GI and Strongyle Parasites in Fecal Samples by Microscopic Examination

The general characteristics of the examined meat goats (*n* = 276) in relation to caprine parasitism are summarized in Table 2. Nine parasite taxa were identified (Supplementary Table S1), including strongyles (67.4%), *Strongyloides papillosus* (5.4%), *Trichuris* spp. (22.1%), *Capillaria* spp. (1.1%), *Moniezia* spp. (1.8%), rumen flukes (18.8%), *Fasciola* spp. (3.3%), *Eimeria* spp. (72.8%), and *Giardia* spp. (0.4%). Additionally, the overall prevalence of GI parasitic infections was 88.8% (*n* = 245/276), with strongyle infections found in 67.4% (*n* = 186/276) and zoonotic infections in 71.0% (*n* = 196/276) of the goats. The prevalence of total GI parasites during the wet season was 45.3%, while strongyle infections were at 35.9%. These figures were comparable to those observed in the dry season, where total GI parasites were present at 43.5% and strongyle infections at 31.5% (Table 2).

**Figure 2:**
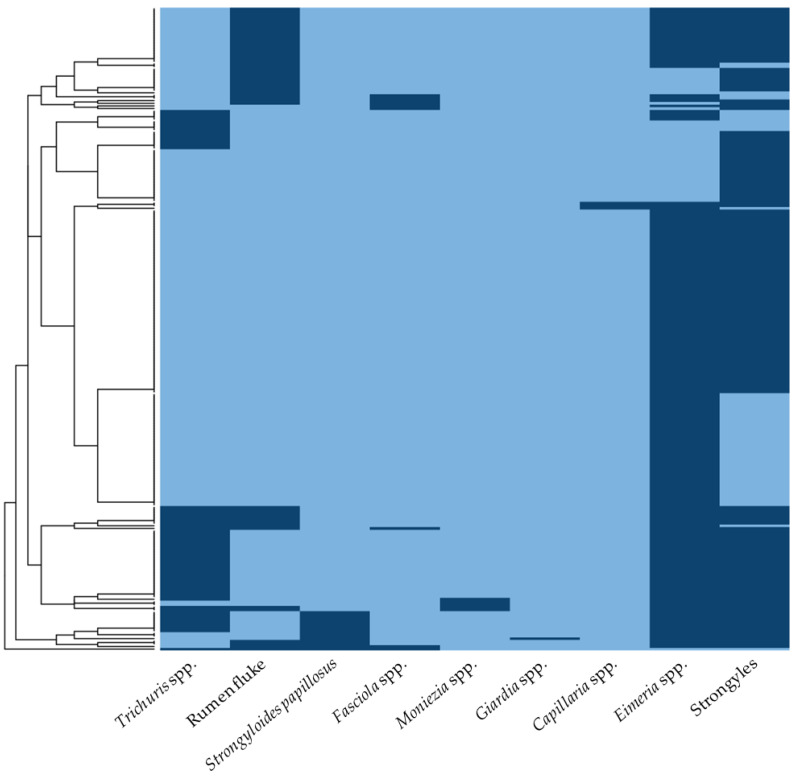
The heat map illustrating the prevalence patterns of parasitic infections in meat goats from southern Thailand. The columns represent individual parasitic infections, while the rows correspond to fecal positive samples. Dark-blue blocks indicate the occurrence of parasites, whereas light-blue blocks signify non-occurrence.

The diversity of GI parasites identified in the goats is presented in Supplementary Table S2. Of the 245 positive samples, 27.8% (*n* = 68) exhibited single infections, while 72.2% (*n* = 177) had mixed infections involving two to five parasite species. The prevalence of double, triple, quadruple, and quintuple infections was 38.4% (*n* = 94), 23.7% (*n* = 58), 8.6% (*n* = 21), and 1.6% (*n* = 4), respectively. Additionally, the prevalence patterns of parasitic infections were represented as a heat map, with dark-blue blocks indicating an occurrence and light-blue blocks indicating a non-occurrence (Figure 2). The hierarchical clustering analysis grouped the sample sets based on occurrence patterns across nine parasites. The elbow plot illustrates the total within-cluster sum of squares as a function of the number of clusters, indicating that four clusters (k = 4) effectively minimize the within-cluster variability (Supplementary Figure S1).

### 3.2. Infection Intensity of Strongyle-Type Eggs and Eimeria oocysts in Relation to Season

As presented in Table 3, the fecal egg counts of strongyle nematodes during the wet season showed the prevalence of low, medium, and high degrees of infection at 29.9%, 9.0%, and 29.9%, respectively. Regarding oocyst counts of *Eimeria* spp., low, medium, and high infections were detected in 59.7%, 10.4%, and 2.1%, respectively. For the dry season (Table 3), low, medium, and high infections of strongyle nematodes were 34.8%, 16.7%, and 12.9%, respectively; while the low, medium, and high infections of *Eimeria* spp. were found at 67.4%, 3.8%, and 1.5%, respectively. The statistical analysis of the infection intensity of strongyle and *Eimeria* parasites in relation to the season indicated a significant association between the strongyle infection intensity and season (Chi-square test = 13.229, df = 3, *p* = 0.004). In contrast, the infection intensity of *Eimeria* spp. was not significantly associated with the season (Chi-square test = 4.950, df = 3, *p* = 0.176) (Table 3).

### 3.3. Risk Factors Associated with Infections of GI Parasites

The associations between total GI parasitic infections and veterinary records are shown in Table 4. The univariable logistic regression analysis identified significant factors associated with the risk of total GI parasitic infections, including gender, BCS, and grazing with other herds. In the multivariable model, female goats and those grazing with other herds demonstrated a significantly higher risk of GI parasitic infections (*p* < 0.05). Focusing on strongyle infections, the univariable logistic regression analysis identified significant factors, including gender, PCV, breed, grazing with other herds, and the grazing rotation (Table 5). In the multivariable model, female goats and those with anemia were at a significantly higher risk of strongyle infection (*p* < 0.05). Conversely, mixed-breed goats demonstrated a protective effect against infection. Detailed associations are provided in Table 5.

### 3.4. Associations Among Clinical Parameters (BCS, FAMACHA, and PCV) and Strongyle EPG

A weak negative correlation was detected between the strongyle EPG and PCV (*r_s_* = −0.353, *p* < 0.001), and a weak positive correlation was found with the FAMACHA score (*r_s_* = 0.397, *p* < 0.001). No significant correlation was found between the strongyle EPG and BCS (*r_s_* = −0.052, *p* = 0.394). Additionally, the PCV showed a strong negative correlation with the FAMACHA score (*r_s_* = −0.858, *p* < 0.001).

### 3.5. Identification of Strongyle Nematode L3 by Microscopic Examination

**Figure 3:**
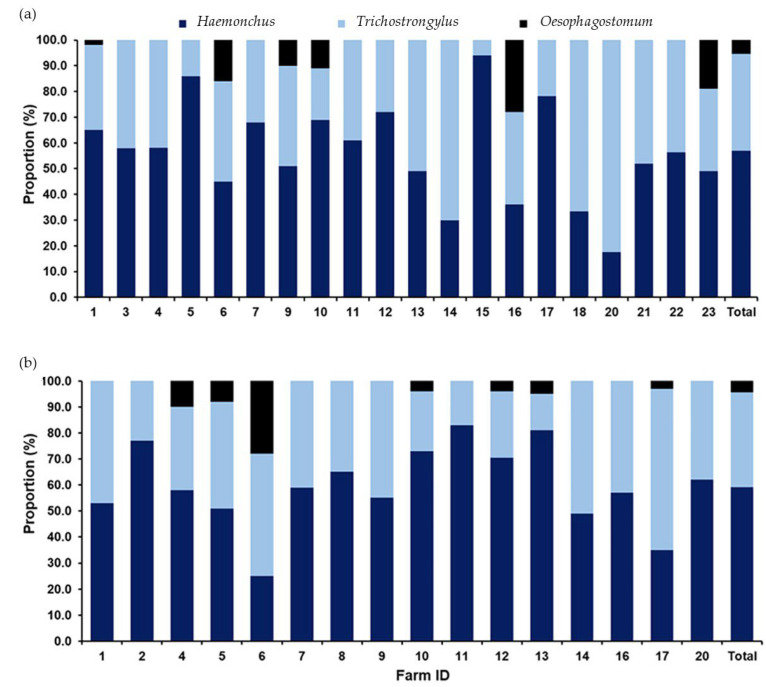
A bar graph illustrating the occurrence of strongyles identified through fecal sample cultures collected from caprine farms in the tropical monsoon climate of southern Thailand. The proportions of strongyle genera are shown for fecal samples collected during (**a**) the wet season and (**b**) the dry season. Dark-blue sections represent *Haemonchus*, light-blue sections indicate *Trichostrongylus*, and black sections denote *Oesophagostomum*.

A total of 2391 L3 of strongyle nematodes collected from 36 positive farms identified three genera: *Haemonchus*, *Trichostrongylus*, and *Oesophagostomum*. Additionally, *Haemonchus* was the predominant genus, comprising 58.0% (*n* = 1700), followed by *Trichostrongylus* at 37.0% (*n* = 1085) and *Oesophagostomum* at 5.0% (*n* = 146). The distribution of each genus across farms during the wet and dry seasons is illustrated in Figure 3, highlighting the seasonal variations in the strongyle prevalence and the potential implications for management practices in livestock health.

### 3.6. Identification of Strongyle Eggs and L3 by Semi-Nested PCR

**Figure 4:**
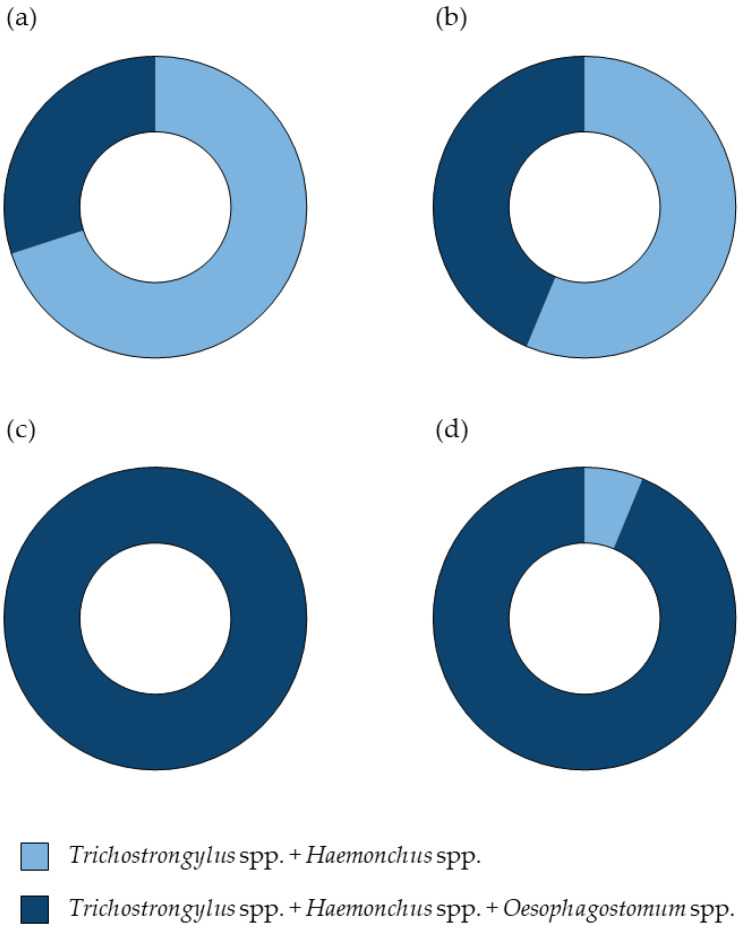
Pie charts illustrating the percentage of the strongyle nematode detection through the microscopic examination and semi-nested PCR. Panel (**a**) represents the wet season results, while panel (**b**) shows the dry season results from the microscopic examination of L3. Panel (**c**) depicts the wet season results, and panel (**d**) presents the dry season results from the semi-nested PCR of strongyle nematode eggs. Dark-blue segments indicate the presence of all three genera, *Haemonchus*, *Trichostrongylus*, and *Oesophagostomum*, whereas light-blue segments signify a co-infection with *Haemonchus* and *Trichostrongylus*.

Supplementary Table S3 summarizes the findings of *Haemonchus*, *Trichostrongylus*, and *Oesophagostomum* from pooled positive fecal samples of individual farms detected by the microscopic examination of L3 and the semi-nested PCR of strongyle nematode eggs during the wet and dry seasons. Regarding strongyle egg samples, mixed infections involving all three genera were detected in 100% of samples during the wet season and 93.7% during the dry season. In contrast, the co-infection with *Haemonchus* and *Trichostrongylus* was observed in only 6.3% of samples during the dry season. All strongyle L3 identified based on morphological characteristics from each farm were accurately confirmed using the semi-nested PCR. Additionally, Figure 4 illustrates the comparative results, highlighting the consistency between the two diagnostic methods.

**Figure 5:**
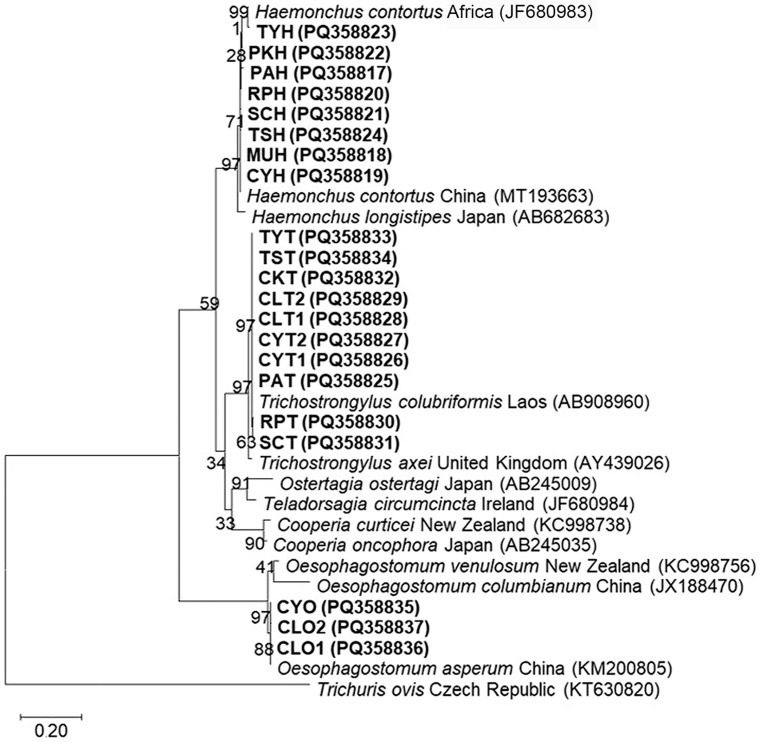
The maximum likelihood tree of ITS2 sequences constructed using the Tamura 3-parameter model with a gamma distribution. Boldface letters denote sequences obtained in this study, including the voucher code and GenBank accession number. The scale bar of 0.20 indicates the evolutionary distance divergence.

A total of twenty-one representative samples, including eight *Haemonchus* samples, ten *Trichostrongylus* samples, and three *Oesophagostomum* samples, were subjected to DNA sequencing. The BLAST search (Supplementary Table S4, including references [35,36,37]) identified eight sequences of *Haemonchus* spp. as *H. contortus* (MT193663 and JF680983) with a 98.82–100% similarity. Ten sequences of *Trichostrongylus* spp. were identified as *Trichostrongylus colubriformis* (AB908960) with a 99.42–100% similarity, while three sequences of *Oesophagostomum* spp. were identified as *Oesophagostomum asperum* (KM200805) with a 99.72–100% similarity. The phylogenetic analysis using the maximum likelihood method demonstrated that all sequences obtained in this study were correctly clustered with their respective species and were clearly separated from other species (Figure 5).

## 4. Discussion

This study provides current data on the GI parasite prevalence in Nakhon Si Thammarat province, filling research gaps over the past decade. Furthermore, the results regarding the risk factors for overall GI and strongyle infections in meat goats may contribute to the development of effective control strategies and help mitigate economic losses.

The overall prevalence of GI parasites in this study was 88.8%, which was lower than that observed in earlier studies in Khon Kaen (100%) [16], Saraburi (96.37%) [6], and the southern area (97.33%) [15]. Conversely, it was higher than prevalence rates documented in other regions of Thailand, such as the northern region (87.2% in Phitsanulok) [14], the central region (68.65% in Bangkok, 79.47% in Nakhon Pathom, 86.54% in Ratchaburi, and 81.86–88% in Kanchanaburi and Saraburi) [8,10,11,12,13], and the southern region (75.83–76.4%) [7,9]. This study also found that goats had a lower GI parasite prevalence than in Myanmar (96%) [38] and China (91.6%) [39] but a higher prevalence than in South Africa (37.1%) [40], Egypt (50.24%) [41], Bangladesh (62.1%) [42], Malaysia (78.6%) [43], Laos (73.96%) [44], Pakistan (82.43%) [45], and Peru (87.80%) [46]. The high prevalence of strongyles and *Eimeria* spp. observed in this study is in agreement with previous findings in Thailand [13,14,15,16] and other countries [38,39,43,44,46]. Furthermore, the low prevalence of *Giardia* spp. (0.4%), *S. papillosus* (5.4%), *Trichuris* spp. (22.1%), *Capillaria* spp. (1.1%), *Moniezia* spp. (1.8%), rumen flukes (18.8%), and *Fasciola* spp. (3.3%) observed in this study is in agreement with previous reports [12,13,15,39]. This study found variations in the prevalence and diversity of GI parasites in Thailand and other countries, which may be attributed to factors such as the geographical region, climate, seasonal variation, sample size, sampling period, identification techniques, and rearing and management practices [15,16,39,42,44].

The present study detected *Haemonchus*, *Trichostrongylus*, and *Oesophagostomum*, which are prevalent infections in goats [13,14,16,47,48]. *Haemonchus* was the dominant genus, accounting for 58.0% of samples, followed by *Trichostrongylus* at 37.0% and *Oesophagostomum* at 5.0%. Comparable larval populations have been documented in other research projects, including those from Malaysia [47], Sudan [49], and Brazil [50]. Most of the previous research consistently identified *Haemonchus* and *Trichostrongylus* as the two most predominant genera, followed by other genera such as *Oesophagostomum* and *Cooperia* [14,16,41,47,48]. A comparison of semi-nested PCR results for strongyle eggs with the larval identification from fecal cultures in the present study revealed differences in the genus composition. In addition, *Oesophagostomum* was not detected in the larval cultures, resulting in a higher prevalence of co-infection with *Haemonchus* and *Trichostrongylus* compared to mixed infections involving all three genera, as identified by the semi-nested PCR targeting strongyle eggs. These differences may be attributed to variations in egg production among female nematodes [51,52] and differences in larval survival rates in the culture, which can vary among parasite species [53,54].

Based on the DNA sequencing, all *Haemonchus*, *Trichostrongylus*, and *Oesophagostomum* sequences obtained in this study were identified as *H. contortus*, *T. colubriformis*, and *O. asperum*, respectively. Similar findings were reported in central (Kanchanaburi), northeastern (Khon Kaen), and northern (Chiang Mai and Lampang) Thailand, where *H. contortus* and *T. colubriformis* were detected [13,16,48]. In contrast to the present study, Income et al. [13] and Rompo et al. [48] detected *Oesophagostomum columbianum* instead of *O. asperum*. *Oesophagostomum venulosum*, *O. columbianum*, and *O. asperum* are regarded as the primary *Oesophagostomum* species infecting sheep and goats, with *O. columbianum* being the most widespread [55,56,57,58]. To date, the global distribution of *O. asperum* has been documented in several countries, including Japan [59], China [37], India [56], Nepal [60], Bangladesh [57], Grenada [58] and, as indicated by the findings of this study, Thailand.

The multivariate analysis performed in this study demonstrated that the risk of overall GI parasite infections was influenced by gender and grazing with other herds, while the risk of strongyle infections was significantly influenced by the gender, breed, and PCV. Male goats were at a lower risk of GI and strongyle infections than female goats. This observation may be influenced by the disparate sample sizes in the present study, where the number of males (*n* = 14) was markedly lower than that of females (*n* = 262). These findings are consistent with earlier findings from Thailand [10,13,16] and other countries [41,43,61]. This finding, however, contradicts earlier studies conducted in Thailand [9,11,12,14] and other countries [44], which reported no significant difference in the prevalence of GI parasites between male and female goats. Moreover, some studies have revealed a greater prevalence of GI infections in male goats than in females [62]. This study found that female goats and those with anemia had an increased susceptibility to strongyle infections, supporting previous studies [16,43,63] that identified a substantial correlation between strongyle infections and reduced PCV levels, indicating anemia in the affected goats. Additionally, this study revealed that mixed-breed goats (*n* = 266) exhibited a greater resistance to strongyle infections than purebred goats (*n* = 10). However, this observation may be influenced by the unequal sample sizes in the present study or by genetic factors governing the host resistance to parasitic infections. In this study, purebred goats included Anglo-Nubian (*n* = 1), Boer (*n* = 7), and Kalahari Red (*n* = 2), while mixed-breed goats resulted from crosses between the southern Thai indigenous breed and Boer (*n* = 245), Anglo-Nubian (*n* = 7), Kalahari Red (*n* = 9), and Saanen (*n* = 5). The observed resistance of mixed-breed goats to strongyle infections could result from their adaptive evolution in an environment where strongyles are common. Conversely, purebred goats, which are exotic breeds utilized for crossbreeding, may lack the same degree of resilience. This finding corresponds with the study conducted by Pralomkarn et al. [64] that found Thai native (TN) goats had greater resistance to *H. contortus* compared to Anglo-Nubian crossbred goats, likely due to their evolutionary adaptation in the prevalent parasite environment. Variations in the resistance to *H. contortus* and GI nematode infections, both within and among goat breeds, have been documented in previous studies [65,66,67,68].

This study reveals a significant negative correlation between strongyle EPG counts and the PCV, suggesting that goats with elevated EPG counts showed significantly lower PCV levels. This finding concurs with previous reports [16,43], indicating that strongyle nematodes, especially *Haemonchus* and *Trichostrongylus*, lead to progressive anemia and hypoproteinemia in small ruminants as a result of chronic intestinal hemorrhage and direct blood-feeding [69]. Furthermore, a strong negative correlation between the PCV and FAMACHA scores was observed in this study, suggesting goats with higher FAMACHA scores generally have lower PCV levels, thereby supporting the FAMACHA scoring system for targeted selective treatment. FAMACHA scores of 1–2 indicate that an anthelmintic treatment is not promptly required, whereas scores of 3–5 denote an urgent need for intervention. No significant correlation between the BCS and strongyle EPG counts was observed in this study, suggesting that the BCS is not a trustworthy indicator for treatment decisions, as 55.1% of goats had a low BCS due to widespread undernutrition. Consequently, we recommend prioritizing treatment for goats exhibiting poor BCSs with FAMACHA scores ranging from 3 to 5, in accordance with the recommendations proposed by Rerkyusuk et al. [16].

Four zoonotic parasites, namely *Giardia* spp., *H. contortus*, *T. colubriformis*, and *Fasciola* spp. [17,19,20,70,71,72,73,74,75,76,77,78], were detected in this study, suggesting a considerable threat to public health. *Giardia* spp. cause giardiasis, characterized by asymptomatic presentations or symptoms such as diarrhea, abdominal cramps, and nausea in humans, acquired by ingesting infective-stage cysts from contaminated food or water or through direct contact with infected humans or animals [72]. *H. contortus* and *T. colubriformis* cause trichostrongylosis, which can lead to asymptomatic conditions, abdominal complications, and hypereosinophilia, primarily due to the ingestion of infective third-stage larvae (L3) from contaminated vegetables or water [71,73,74,75,76]. *Fasciola* spp. was a zoonotic liver fluke, causing human fascioliasis, whose common symptoms are epigastric pain, upper abdominal pain, and malaise, primarily transmitted through the ingestion of metacercariae-contaminated freshwater plants and drinking water with metacercariae [70,77,78]. Considering the public health implications, the government should promote health education, good personal hygiene practices, and the implementation of appropriate parasite control measures for farmers or personnel who are in close contact with goats. To mitigate environmental contamination, infected animals should be rapidly treated to reduce the parasite burden and transmission, and the use of fresh goat feces as fertilizer should be avoided. Additionally, the routine surveillance and monitoring of zoonotic parasites in goats should be implemented to prevent human health risks.

## 5. Conclusions

*Eimeria* spp. and strongyle nematodes have become the two most prevalent parasites in meat goats, with co-infections involving multiple parasite types and seasonal variations, whereby the wet season corresponded with a higher infection intensity of strongyle and *Eimeria* spp. compared to the dry season. Remarkably, this study is the first report of *O. asperum*’s presence in meat goats in Thailand. Gender and grazing with other herds were important risk factors for overall GI parasitic infections. In contrast, the gender, breed, and PCV significantly affected the risk of strongyle infection. Furthermore, PCV and FAMACHA scores were associated with strongyle EPG counts, indicating the need for effective management strategies aimed at animals with a low BCS and high FAMACHA scores to reduce the harmful effects of strongyle infections on the health and productivity of meat goats. To implement a sustainable parasite control strategy, it is crucial to implement a comprehensive approach that incorporates targeted treatment, pasture management, nutritional supplementation, and selective breeding. In addition, goat farmers should be educated on zoonotic diseases and their prevention and control by the government, including annual deworming, promptly treating infected animals to minimize environmental parasitic contamination, avoiding using fresh goat feces as fertilizer, and ensuring proper vegetable cleaning before consumption.

## Figures and Tables

**Table 1:** Primer sequences for strongyle detection by semi-nested PCR.

Genus	Primer Name	Region	Sequence (5′–3′)	Size of PCR Product (bp)
All strongyles	Strongyle F2	5.8S rRNA	TGGTGAAATTTTGAACGCATAG	324–349
	Strongyle R3	28S rRNA	ATGCTTAAGTTCAGCGGGTA	
*Cooperia*	Cooper R	ITS2	CGAATACTACTATCTCCAACATG	293
*Haemonchus*	Haemo R	ITS2	GTACACTCAAATAGWGGCAACAT	227
*Oesophagostomum*	Oeso R	ITS2	CTCATCTAGAACGAGGATCACA	143
*Trichostrongylus*	Tricho R	ITS2	CAATATTTGAYAATGACCATTCG	128

rRNA: Ribosomal RNA; ITS: Internal Transcribed Spacer.

**Table 2:** General characteristic of examined meat goats (*n* = 276) associated with GI parasitic and strongyle infections in Nakhon Si Thammarat province, southern Thailand.

Variable	Category	Samples (*n*)	No. of Positive Samples (%)	
			GI Parasites	Strongyles
Season	Wet	144	125 (45.3)	99 (35.9)
	Dry	132	120 (43.5)	87 (31.5)
Breed	Pure	10	10 (3.6)	9 (3.3)
	Mixed	266	235 (85.2)	177 (64.1)
Gender	Male	14	7 (2.5)	6 (2.2)
	Female	262	238 (86.2)	180 (65.2)
Age	≤2 years	71	64 (23.2)	47 (17.0)
	>2 years	205	181 (65.6)	139 (50.4)
BCS	Poor	152	139 (50.4)	108 (39.1)
	Good	124	106 (38.4)	78 (28.3)
PCV	Normal	186	162 (58.7)	110 (39.9)
	Anemia	90	83 (30.1)	76 (27.5)
FAMACHA score	1	33	29 (10.5)	16 (5.8)
	2	97	81 (29.3)	51 (18.5)
	3	113	102 (37.0)	89 (32.2)
	4	27	27 (9.8)	25 (9.1)
	5	6	6 (2.2)	5 (1.8)
Farm management	Intensive	192	162 (58.7)	106 (38.4)
	Semi-intensive	84	83 (30.1)	80 (29.0)
Water source nearby	No	113	95 (34.4)	73 (26.4)
	Yes	163	150 (54.3)	113 (40.9)
Grazing with other herds	No	234	204 (73.9)	148 (53.6)
	Yes	42	41 (14.9)	38 (13.8)
Grazing rotation	No	252	222 (80.4)	166 (60.1)
	Yes	24	23 (8.3)	20 (7.2)
Deworming interval	≤6 months	258	227 (82.2)	174 (63.0)
	>6 months	18	18 (6.5)	12 (4.3)
The number of drug types used per year	1 type	102	91 (33.0)	65 (23.6)
	2 types	174	154 (55.8)	121 (43.8)
Total		276	245 (88.8)	186 (67.4)

GI: Gastrointestinal; BCS: Body condition score; PCV: Packed cell volume; EPG: Eggs per gram; and *n*: No. of samples.

**Table 3:** The infection intensity of strongyle-type eggs and *Eimeria* spp. in meat goats during the wet season (*n* = 144) and dry season (*n* = 132).

Parasite	Degree of Infection	Wet Season		Dry Season		*p*-Value
		Samples (%)	Mean ± SD	Samples (%)	Mean ± SD	
Strongyles ^#^						
	Negative	45 (31.3)	-	47 (35.6)	-	0.004
	Low	43 (29.9)	123.26 ± 103.13	46 (34.8)	189.13 ± 138.21	
	Moderate	13 (9.0)	642.31 ± 153.9	22 (16.7)	734.09 ± 186.69	
	High	43 (29.9)	2441.86 ± 1449.18	17 (12.9)	1502.94 ± 415.13	
*Eimeria* spp. ^‡^						
	Negative	40 (27.8)	-	36 (27.3)	-	0.176
	Low	86 (59.7)	465.12 ± 452.64	89 (67.4)	470.22 ± 478.08	
	Moderate	15 (10.4)	3240.0 ± 992.69	5 (3.8)	2190.0 ± 207.36	
	High	3 (2.1)	14,250.0 ± 5239.51	2 (1.5)	12,700.0 ± 5850.0	

EPG: Eggs per gram; OPG: Oocysts per gram; SD: Standard deviation. ^#^ Classification of strongyles: Negative, Low (<500 EPG), Moderate (500–1000 EPG), High (>1000 EPG). ^‡^ Classification of *Eimeria* spp.: Negative, Low (<1800 OPG), Moderate (1800–6000 OPG), High (>6000 OPG).

**Table 4:** Factors associated with the positivity of the total GI parasitic infection in meat goat farms based on univariable and multivariable logistic regression analyses at the animal level (Hosmer and Lemeshow test *p*-value = 0.06).

Logistic Regressions	Variable	Category	Control	Case	*p*-Value	OR	95%CI
Univariable							
	BCS	Good	18	106			
		Poor	13	139	0.11	1.81	0.34–2.92
	Gender	Male	7	7			
		Female	24	238	<0.001	9.75	2.71–27.21
	Grazing with other herds	No	30	204			
		Yes	1	41	0.04	6	0.96–86.84
Multivariable							
	Gender	Male	7	7			
		Female	24	238	<0.001	8.57 ^a^	2.71–27.21
	Grazing with other herds	No	30	204			
		Yes	1	41	0.131	4.79 ^a^	0.96–86.84

GI: Gastrointestinal; BCS: Body condition score, OR: Odds ratio; CI: Confidence intervals; ^a^: denotes adjusted odds ratio.

**Table 5:** Factors associated with the positivity of the strongyle infection in meat goat farms based on univariable and multivariable logistic regression analyses at the animal level (Hosmer and Lemeshow test *p*-value = 0.9).

Logistic Regressions	Variable	Category	Control	Case	*p*-Value	OR	95%CI
Univariable							
	Gender	Male	8	6			
		Female	82	180	0.04	2.91	0.85–10.54
	PCV	Normal	76	110			
		Anemia	14	76	<0.001	3.73	1.92–7.69
	Breed	Pure	1	9			
		Mixed	89	177	0.12	0.22	0.004–1.64
	Grazing rotation	No	86	166			
		Yes	4	20	0.12	0.38	0.09–1.21
	Grazing with other herds	No	86	148			
		Yes	4	38	0.001	5.49	1.88–21.91
Multivariable							
	Gender	Male	8	6			
		Female	82	180	0.037	3.56 ^a^	1.10–12.49
	PCV	Normal	76	110			
		Anemia	14	76	<0.001	4.05 ^a^	2.17–8.03
	Breed	Pure	1	9			
		Mixed	89	177	0.088	0.16 ^a^	0.01–0.89

OR: Odds ratio; CI: Confidence interval; PCV: Packed cell volume; ^a^: denotes adjusted odds ratio.

## Data Availability

The data presented in this study are included in the article.

## Supplementary Materials

The following supporting information can be downloaded at https://www.mdpi.com/article/10.3390/ani15142040/s1: Figure S1: The determination of the optimal number of clusters for the prevalence patterns of parasitic infections using the elbow. The elbow plot illustrates the total within-cluster sum of squares (WSS) as a function of the number of clusters (k). The elbow at k = 4 indicates that four clusters effectively minimize within-cluster variability; Table S1: The prevalence of GI parasites among 276 meat goats in Nakhon Si Thammarat province, southern Thailand; Table S2: Th prevalence of the single infection and co-infection of GI parasites of meat goats (*n* = 245) detected by microscopic examination; Table S3: Strongyle genera identified from pooled fecal positive samples of individual farms detected by the microscopic examination of L3 and semi-nested PCR of strongyle nematode eggs during the wet and dry seasons; and Table S4: BLAST searches of strongyle sequences from representatives of known genera of L3.
