# Supplementary material for: Seasonal Infective Dynamics and Risk Factors Associated with Prevalence of Zoonotic Gastrointestinal Parasites from Meat Goats in Southern Thailand
Source: Animals (Basel). 2025 Jul 11;15(14):2040. doi: 10.3390/ani15142040 (PMC12291659; doi:10.3390/ani15142040)
Supplement: Supplementary file 1 [file animals-15-02040-s001.zip › animals-3726021-supplementary.pdf]

Table S1. Prevalence of GI parasites among 276 meat goats in Nakhon Si Thammarat province, southern Thailand.

GI parasite	Stage	No. of positives	Prevalence (%)	Zoonotic status
**Nematode**				
Strongyles	Egg	186	67.4	Zoonotic (some species)
Strongyloides papillosus	Egg	15	5.4	Non-zoonotic
Trichuris spp.	Egg	61	22.1	Non-zoonotic
Capillaria spp.	Egg	3	1.1	Non-zoonotic
**Cestode**				
Moniezia spp.	Egg	5	1.8	Non-zoonotic
**Trematode**				
Rumen flukes	Egg	52	18.8	Non-zoonotic
Fasciola spp.	Egg	9	3.3	Zoonotic
**Protozoa**				
Eimeria spp.	Oocyst	201	72.8	Non-zoonotic
Giardia spp.	Cyst	1	0.4	Zoonotic

GI: Gastrointestinal

Table S2. Prevalence of single infection and co-infection of GI parasites of meat goats (n = 245) detected by microscopic examination.

Parasite infection	No. of positive samples (%)
**Single infection**	
Eimeria spp.	43 (17.6)
Strongyles	20 (8.2)
Trichuris spp.	4 (1.6)
Rumen flukes	1 (0.4)
Total	68 (27.8)
**Double infection**	
Strongyles + *Eimeria* spp.	70 (28.6)
Strongyles + *Trichuris* spp.	7 (2.9)
Strongyles + Rumen flukes	9 (3.7)
Strongyles + *Fasciola* spp.	1 (0.4)
Trichuris spp. + *Eimeria* spp.	4 (1.6)
Capillaria spp. + *Eimeria* spp.	1 (0.4)
Rumen flukes + *Eimeria* spp.	2 (0.8)
Total	94 (38.4)
**Triple infection**	
Strongyles + *Strongyloides papillosus* + *Eimeria* spp.	2 (0.8)
Strongyles + *Trichuris* spp. + *Eimeria* spp.	26 (10.6)
Strongyles + *Capillaria* spp. + *Eimeria* spp.	2 (0.8)
Strongyles + *Moniezia* spp. + *Eimeria* spp.	2 (0.8)
Strongyles + Rumen flukes + *Eimeria* spp.	21 (8.6)
Strongyles + *Fasciola* spp. + *Eimeria* spp.	1 (0.4)
Trichuris spp. + Rumen flukes + *Eimeria* spp.	1 (0.4)
Strongyles + Rumen flukes + *Fasciola* spp.	1 (0.4)
Rumen flukes + *Fasciola* spp. + *Eimeria* spp.	2 (0.8)
Total	58 (23.7)
**Quadruple infection**	
Strongyles + *Strongyloides papillosus* + *Trichuris* spp. + *Eimeria* spp.	8 (3.3)
Strongyles + *Strongyloides papillosus* + Rumen flukes + *Eimeria* spp.	2 (0.8)
Strongyles + *Strongyloides papillosus* + *Giardia* spp. + *Eimeria* spp.	1 (0.4)
Strongyles + *Trichuris* spp. + *Moniezia* spp. + *Eimeria* spp.	1 (0.4)
Strongyles + *Trichuris* spp. + Rumen flukes + *Eimeria* spp.	7 (2.9)
Strongyles + Rumen flukes + *Fasciola* spp. + *Eimeria* spp.	1 (0.4)
Strongyloides papillosus + *Trichuris* spp. + Rumen flukes + *Fasciola* spp.	1 (0.4)
Total	21 (8.6)
**Quintuple infection**	
Strongyles + *Strongyloides papillosus* + Rumen flukes + *Fasciola* spp. + *Eimeria* spp.	1 (0.4)
Strongyles + *Trichuris* spp. + *Moniezia* spp. + Rumen flukes + *Eimeria* spp.	2 (0.8)
Strongyles + *Trichuris* spp. + Rumen flukes + *Fasciola* spp. + *Eimeria* spp.	1 (0.4)
Total	4 (1.6)

GI: Gastrointestinal; n: No. of samples.

Table S3. Strongyle genera identified from pooled fecal positive samples of individual farms detected by microscopic examination of L3 and semi-nested PCR of strongyle nematode eggs during the wet and dry seasons.

Strongyle infection	No. of strongyle detected (%)		
	Wet season (n = 20)	Dry season (n = 16)	Total (n = 36)
**Microscopic examination of L3 larvae**			
Trichostrongylus spp. + *Haemonchus* spp.	14 (70.0%)	9 (56.3%)	23 (63.9%)
Trichostrongylus spp. + *Haemonchus* spp. + *Oesophagostomum* spp.	6 (30.0%)	7 (43.7%)	13 (36.1%)
**Semi-nested PCR of strongyle nematode eggs**			
Trichostrongylus spp. + *Haemonchus* spp.	—	1 (6.3%)	1 (2.8%)
Trichostrongylus spp. + *Haemonchus* spp. + *Oesophagostomum* spp.	20 (100.0%)	15 (93.7%)	35 (97.2%)

L3: Third-stage larvae; PCR: Polymerase chain reaction; n: number of samples

Table S4. BLAST searches of strongyle sequences from representatives of known genera of L3.

Voucher code	Result of sequencing		GenBank ID	Host animal	Country	Reference
	**Species from BLAST top hit**	**% identity to BLAST top hit**				
PAH	*Haemonchus contortus*	98.82	MT193663	Goat	China	[35]
MUH	*Haemonchus contortus*	100.00	MT193663	Goat	China	[35]
CYH	*Haemonchus contortus*	100.00	MT193663	Goat	China	[35]
RPH	*Haemonchus contortus*	99.70	MT193663	Goat	China	[35]
SCH	*Haemonchus contortus*	99.70	MT193663	Goat	China	[35]
PKH	*Haemonchus contortus*	99.41	MT193663	Goat	China	[35]
TYH	*Haemonchus contortus*	98.82	JF680983	Sheep	Africa	Unpublished
TSH	*Haemonchus contortus*	99.70	MT193663	Goat	China	[35]
PAT	*Trichostrongylus colubriformis*	100.00	AB908960	Goat	Laos	[36]
CYT1	*Trichostrongylus colubriformis*	100.00	AB908960	Goat	Laos	[36]
CYT2	*Trichostrongylus colubriformis*	100.00	AB908960	Goat	Laos	[36]
CLT1	*Trichostrongylus colubriformis*	100.00	AB908960	Goat	Laos	[36]
CLT2	*Trichostrongylus colubriformis*	100.00	AB908960	Goat	Laos	[36]
RPT	*Trichostrongylus colubriformis*	99.42	AB908960	Goat	Laos	[36]
SCT	*Trichostrongylus colubriformis*	99.71	AB908960	Goat	Laos	[36]
CKT	*Trichostrongylus colubriformis*	100.00	AB908960	Goat	Laos	[36]
TYT	*Trichostrongylus colubriformis*	100.00	AB908960	Goat	Laos	[36]
TST	*Trichostrongylus colubriformis*	100.00	AB908960	Goat	Laos	[36]
CYO	*Oesophagostomum asperum*	100.00	KM200805	Goat	China	[37]
CLO1	*Oesophagostomum asperum*	100.00	KM200805	Goat	China	[37]
CLO2	*Oesophagostomum asperum*	99.72	KM200805	Goat	China	[37]

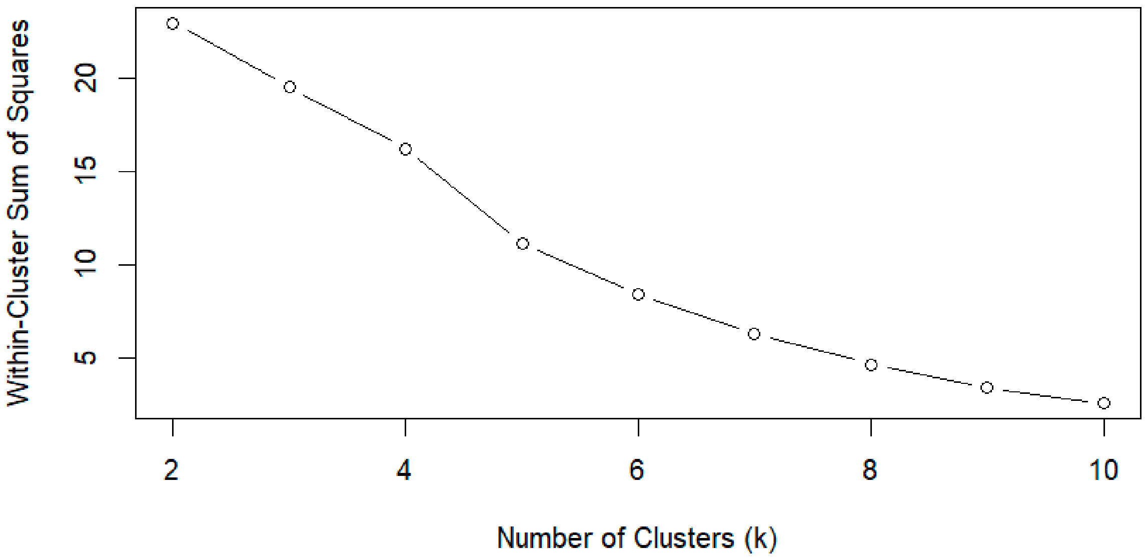

Figure S1. Determination of the optimal number of clusters for the prevalence patterns of parasitic infections using the elbow. The elbow plot illustrates the total within-cluster sum of squares (WSS) as a function of the number of clusters (k). The elbow at k = 4 indicates that four clusters effectively minimize within-cluster variability.
